# An analysis of the gender and social determinants of health in urban poor areas of the most populated cities of Pakistan

**DOI:** 10.1186/s12939-022-01657-w

**Published:** 2022-04-18

**Authors:** Khawaja Aftab Ahmed, John Grundy, Lubna Hashmat, Imran Ahmed, Saadia Farrukh, Dexter Bersonda, Muhammad Akram Shah, Soofia Yunus, Hari Krishna Banskota

**Affiliations:** 1 UNICEF Pakistan, St. 5., Diplomatic Enclave, Islamabad, Pakistan; 2 College of Public Health, Medicine, and Veterinary Sciences, James Cook University, Townsville, Australia; 3 Civil Society Human and Institutional Development Programme – CHIP, CHIP House # 1, Street # 9, G.8/2, Islamabad, Pakistan; 4 UNICEF CO Yemen, Sanaa, Yemen; 5 UNICEF Consultant, Manila, The Philippines; 6 EPI Program, National Institute of Health (NIH), Prime Minister’s Health Complex, Park Road, Chakshahzad, Islamabad, Pakistan; 7 UNICEF, Pakistan, St. 5., Diplomatic Enclave, Islamabad, Pakistan

**Keywords:** Gender, Immunization, Primary health care, Vaccination, Social determinants, Pakistan

## Abstract

**Background:**

Recent surveys, studies and reviews in urban areas of Pakistan have highlighted the impacts of social inequities on access of women and children to health services for women and children in Pakistan.

**Objectives:**

The Urban Slum Profiles and coverage surveys were conducted between 2017 and 2019. The objective of the profiles was to obtain an updated listing of slums and other underserved areas, and to better understand current vaccination and health service coverage in these areas. Utilising findings from these studies, this paper aims to better understand the gender and social determinants of health that are giving rise to health inequalities in the slums.

**Methods:**

The Urban Slum Profiles adopted a mixed methods approach combining both qualitative and quantitative methods. The study was comprised of two main survey approaches of Urban Slum Profiles and Immunisation Coverage Survey in 4431 urban poor areas of the 10 most highly populated cities of Pakistan.

**Results:**

Findings are classified into six analytic categories of (1) access to health services, (2) female workforce participation, (3) gender-friendly health services, (4) access to schools and literacy, (5) social connections, and (6) autonomy of decision making. Out of a national sample of 14,531 children in urban poor areas of 10 cities, the studies found that just over half of the children are fully immunised (54%) and 14% of children had received zero doses of vaccine. There are large shortages of health facilities and female health workforce in the slums, with significant gaps in the quality of health infrastructure, which all serve to limit both demand for, and supply of, health services for women and children. Results demonstrate low availability of schools, low levels of female literacy and autonomy over decision making, limited knowledge of the benefits of vaccination, and few social connections outside the home. All these factors interact and reinforce existing gender norms and low levels of health literacy and service access.

**Conclusion:**

The Urban Slum profiles and coverage studies provide an opportunity to introduce gender transformative strategies that include expansion of a female health workforce, development of costed urban health action plans, and an enabling policy environment to support community organisation and more equitable health service delivery access.

## Background

In recent years, as national health care systems across the globe step towards eradication and elimination targets for Vaccine Preventable Diseases (VPDs), increased policy and planning attention is being given to reaching the last 10-15% of unvaccinated or partially vaccinated populations, with an estimated 19.4 million of the world’s children not receiving their basic vaccines in 2019 [1].Multiple surveys across the globe show that this last segment of unreached population is often exposed to social or economic disadvantage, or are residing in urban poor [2–4] or remote geographic locations [5].

The impact of gender on uptake of vaccination has been illustrated in several country studies. Analysis from three National Family Health Surveys in India between 1992 and 2006 found that girls were found to have significantly lower immunization coverage than boys [6].A recent study in rural Bangladesh in 2018 found that there is a significant variation in coverage according to the sex of the child in favour of the boys (89.2% versus 85.9% for girls) [7]. In Pakistan, national surveys have demonstrated that there is a significant gap between the rate for fully immunised child of for males (68%) than for females (63%) [8]. In addition, lower status in the household and community, barriers relating to social position (including economic status, ethnicity, marital status, age, educational status, socio-cultural context), gender norms, levels of health literacy, and women’s experience of quality of services have been identified as additional key gender-related barriers to immunization [9]. Women, as primary care givers, require autonomy of decision making to ensure that both themselves and their children (both boys and girls) can readily access and utilise primary health care services. Autonomy of decision making also assumes a higher level of knowledge and participation by fathers in health and education. In several international and national settings, studies and reviews have demonstrated that such autonomy is compromised by socio cultural factors and gender norms [10–12]. After controlling for socioeconomic variables, studies in Nigeria have found that household decision-making and domestic violence behaviours were significantly associated with a child being fully immunized [13]. This is consistent with the findings of previous studies in Pakistan, that validate the lack of female autonomy over health care decision making, especially in rural areas and in urban slums, and how this limits their access to health care [14, 15]. A review of gender and health policy in Pakistan found that the fact that women had little decision making power about using health care services was due to women living within a set of societal norms, which preferences males for decision making, and places women in a subordinate position, all of which serve to increase vulnerability to illness [16].

The reasons for these gender inequities involve complex interactions between health sector specific determinants as well as broader social influences on health seeking behaviours [17]. These factors include education level, economic and political participation, and advocacy for reproductive health rights [18]. This complexity in the gender related determinants of health is reflected in research findings in Pakistan on the economic, environmental, and social exposures related to low immunisation coverage. A cross sectional study of measles vaccination in one district in Pakistan found that having an educated mother, discussing vaccinations, having correct knowledge about vaccinations, living closer to health facilities and housing conditions were all factors associated with increased use of vaccination services [19]. A review of the impact of early marriage found that it was associated with lower use of ante natal care and delivery assistance from skilled persons [20].

The nexus between gender and access to primary health care (PHC) services has been elaborated in several PHC studies in Pakistan between 2017 and 2021. These studies confirm gender-related barriers to PHC services in relation to facility locations, distance, transport, staff availability, income, service hours, and service organisation [21]. A study of family planning services in Balochistan province concluded that the main enabling factors for access included availability of a female doctor, the knowledge of lady health visitor, availability of contraceptives and the quality of services [22]. One other study of health service utilisation of PHC services in rural Pakistan found gender differences in satisfaction levels, with women’s main concerns related to accessibility to health facilities and to reproductive health care, whereas men’s concerns included responsiveness and presence of staff and services hours. The latter study also highlighted the role of cultural factors in reducing accessibility to health facilities, as travel to health facilities by women alone may be restricted by perceptions of what is respected or safe in local culture [23]. These studies suggest a complex interaction between gender, social and health system factors that shape patterns of health care access and utilisation in Pakistan.

Inequities in access to education means that health literacy can be gendered, in that limited understanding of immunisation reduces motivation to seek out vaccination services and negotiate the pathways of health system [9]. These complex interactions are also reflected in health outcomes, with some studies demonstrating that doubling the proportion of girls educated at the secondary level reduces the fertility rate by 1.4 children per women and reductions in infant mortality rates by between five and 10 % for each additional year in a mothers schooling [18]. This relationship between health and education is also inverse, with immunisation coverage contributing to improved growth and educational achievement in children [24]. These interactions and feedback loops between gender, health and society are characteristics of complex adaptive systems, which challenges policy-makers and planners to identify policy and planning points of engagement [25] so as to reset the gender norms that currently set such rigid boundaries for the health seeking behaviours of households.

Utilising the findings from immunization coverage surveys in slums [26] and urban slum profiles [27] in the 10 largest cities of Pakistan, this paper examines the extent to which gender is a determinant of frontline health care (PHC) service access and coverage for the urban poor in these ten cities. Although the main health focus of service access and coverage in these Urban Slum Profiles relates to immunisation and frontline health services in the slums, the complex interactions between economic, social and gender determinants of health care access illustrate the potential these findings have for broader primary health care (PHC) policy and practice.

The objective of the profiles was to obtain an updated listing of slums and other underserved areas in urban poor areas of Pakistan, and to better understand current vaccination and health service coverage in these areas, and to set a baseline for development of urban health initiatives. Utilising findings from these studies, this paper aims to better understand the gender and social determinants of health that are giving rise to health inequalities in the slums.

## Methods

### Approach

The Urban Slum Profiles and coverage surveys were conducted between 2017 and 2019. The study adopted a mixed methods approach combining both qualitative and quantitative methods. The study was comprised of two main survey approaches of Urban Slum Profiles and Immunisation Coverage Survey of Urban Poor Areas in 10 cities of Pakistan. .

### Profile of study areas

The urban slums were identified in accordance with the international definition of slum areas, and underserved areas were identified by surveyors and provincial managers, based on criteria of low immunisation coverage and high rates of vaccine refusal. A total of 4431 areas were identified in the 10 cities,^1^ of which 3031 were slums, with a total population of 19.6 million (comprising 48% of the total urban population of over 41 million).

### Study design

There were four types of assessment that made up the urban slum profiles and included physical verification of slums and underserved areas, assessment of EPI Facilities (*n* = 422), mapping of coverage areas and assessing the level of health resources in the union councils (UCs).^2^ The health profiles were complemented by a national immunisation coverage survey in the urban poor areas of 10 cities. The immunization coverage survey in slums and underserved areas of 14,491 mothers and 14,531 children and 14,467 households in 10 cities was conducted according to the protocols of the World Health Organisation on immunisation coverage surveys [28]. These surveys also collected data on knowledge of vaccination and health care services, which was triangulated with the urban slum profile assessments and focus groups discussions conducted in the field on the topics of immunisation and health services access. Coverage survey questionnaires covered topics including physical features of the houses, economic situation of the households, profile of mothers, and the status of vaccination.

### Sampling and study participants

For the health profiles the list of slums and underserved areas was prepared, each UC was individually visited by the survey team to verify the slum areas. Following physically verification of the areas, information for a social profile was collected through group discussions with people belonging to those areas. The study team conducted one group discussion from each union council located in the slums and underserved areas. Three to five respondents were selected based on inclusion and exclusion criteria for the interactive group discussions. The inclusion criteria included persons who has been living in the area for more than 2 years, and who had some knowledge of the physical infrastructure and other facilities of that area. Residents who resided in the areas for less than 2 years were excluded. The EPI facility surveys were conducted using a structured questionnaire. EPI facilities surveyed in or near slums & underserved areas were physically assessed for availability, numbers and types of services, human resources, availability of vaccines & supplies, cold chain, waste management, water and sanitation at facility. All EPI facilities located in the slums and underserved areas were assessed (*n* = 422). In addition to physical verification, social profiling and facility surveys, consultations were undertaken at the District level in each city for providing official information on health resources in the survey areas.

### Study limitations

These profiles and coverage surveys were conducted as part of internal program evaluation and were conducted under the stewardship of the Provincial Expanded Program of Immunisation (EPI), local government and civil and international agencies. Gender, although not the primary survey objective, was mainstreamed into survey design, particularly in relation to establishing associations between health coverage and the background characteristics of households. As an internal method of evaluation designed to improve program and health system performance, this research combines both quantitative and qualitative research. It can be best categorised as a model of “implementation research,” whose principal concern is how interventions work in real world settings [29]. One of the main limitations of this research relates to the need to “embed” research within implementing institutions [30], to increase the probability for take up of recommendations into policy and practice. This raises the risk that data collection can be subject to systematic bias. Researchers and implementers tried to mitigate this risk through triangulation of findings from multiple data sources including from quantitative coverage survey methods, focus group discussion with community members, and physical verification of slum areas and facilities.

### Approach to analysis

The approach to the gender and social determinants analysis was guided by searching through the urban slum profile reports and through examination of coverage survey data bases for gender related data, analysis, commentary, or recommendations. The design of the survey tools facilitated this approach as the urban slum profiles were constructed to provide a social determinants of health perspective, through examination of information on demography, infrastructure, social services, health services and assessments of health resources in the slums, as well as through examination of background characteristics of households in the coverage survey. (See Table 1).

**Table 1:** Summary of Study Types and Sample Sizes

Study Types	Data Type	Sample Sizes	# Cities
Urban Slum Profile - Physical verification	Slums & underserved areas were physically verified, and social profile collected through group discussions.	868 Union Councils	10
Urban Slum Profile - EPI Facility Surveys	EPI facilities physically assessed for availability, numbers and types of services, human resources, availability of vaccines & supplies, cold chain, waste management, water and sanitation at facility.	422 Facilities	8
Urban Slum Profile - Resource Mapping	UC maps were prepared with locations of slums, underserved areas. Health facilities were recorded through real time Geographic Information System (GIS) coordinates.	868 Union Councils	10
Urban Slum Profile Resource Assessment	Data of health resources available at UC level in relation to facilities, human resources, availability of EPI services	868 Union Councils 4441 Slums & Underserved Areas	10
Immunisation Coverage Surveys	Survey applied the WHO cluster survey methodology with data collected on immunisation coverage for multiple antigens and for card retention, and background characteristics of households including education levels, livelihoods, housing, environmental conditions and knowledge about vaccination.	14,531 Children, 14,491 Mothers, 14,467 Households	10

**Fig. 1:**
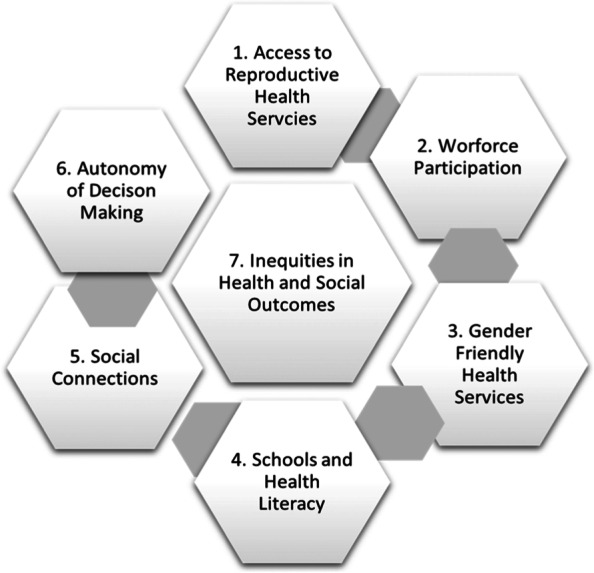
Classification of Gender Related Information Urban Slum Profiles Pakistan 2017-2019

Figure 1 was constructed following extraction of information on gender from the urban slum profile reports and survey data and classifies gender related information in these profiles according to the seven categories.

This framework provides a means by which to classify and describe the information (refer to findings section), as well as a means by which to examine interactions and feedback loops between the different categories, which all reflect the lived experiences and social conditions of women and their families in the slums.

Some elements of gender theory include how gender norms are socialised through inequitable power relations between men and women, and how rules of governing institutions and social interaction both reflect and reinforce gender norms. Both of these perspectives will be applied in relation to discussion of main policy and planning implications of findings in the concluding section [31].

## Results

### Availability of health services

These inequities in reproductive health care access relate not only to gaps in provision of types of services, but also to availability of facilities. Overall, of the UCs where health resource assessments were conducted (*n* = 636), 26% (*n* = 165) are without EPI Facilities, and 31% (*n* = 197) are without any health facilities at all (whether public or private).

Fixed facilities are a requirement for most reproductive health services. Only 3% (*n* = 232) of the slums and underserved areas out of a total of 4431 areas in ten cities are located within a 3 km distance from the slum areas, and 29% (*n* = 1285) of the slum and underserved areas do not receive health outreach services from fixed facilities. Limited transport also impedes accessibility. In Quetta City of Balochistan Province, the Urban Slum Profile reports that access by female caregivers to health facilities is limited by the fact that there are scattered residential areas which are devoid of gender-friendly transport facilities [32]. In Karachi, with a population of 7,645,375, only 9% of the public health facilities located in the slums have an ambulance service. Although trends in facility delivery are improving (66% in 2017-2018 compared to 13% in 1990-91), it remains the case that 1 in 3 births are still occurring at home [33].

### Female workforce participation

In the context of gender and health, workforce participation can refer to participation by women in the general workforce generally or in the health workforce specifically. In collecting data on the household characteristics in the national coverage survey, information was sought on the current employment status of women and men in the households, with 14,491 women mothers being interviewed in the slums and underserved areas of 10 cities.

**Fig. 2:**
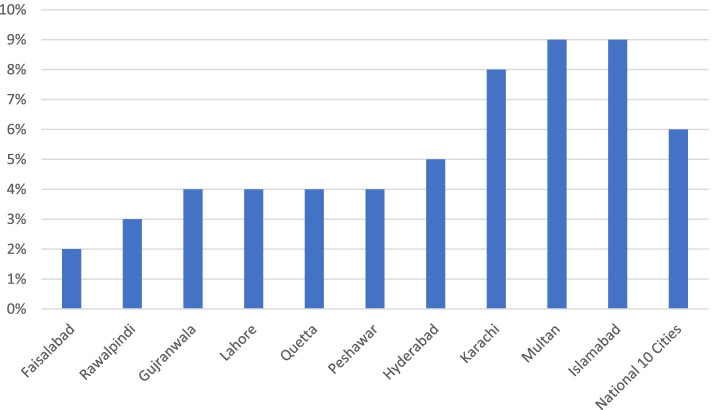
Percentage of Mothers with Employment Outside the Home in Urban Slums and Underserved Areas in 10 Cities

Nationally, of the 14,491 women surveyed in 4431 slum and underserved areas in 10 cities, only 6% (*n* = 833) had employment outside the home. Workforce participation rates for mothers were as low as 3% in Rawalpindi (*n* = 26 of 824 respondents) and 2% in Faisalabad (*n* = 13 out of 712 respondents) Fig. 2.

This low workforce participation rate may be partly because the mothers interviewed all had children between ages 12 months and 23 months (the age parameters for inclusion in the immunisation coverage survey).

The association between low female workforce participation and socio-economic status are reinforced by the findings of the immunization coverage survey in slums and underserved areas. Of the 14,467 households surveyed, 54% (*n* = 7846) of households derive incomes from daily wages and 56% of households (*n* = 8060) are either occasionally or are always in income deficit. Just 6% of households (*n* = 888) experience income surplus (i.e. have household savings).

One opportunity presented in the Urban Slum Profiles for improvement in health and social conditions is increased participation by members of the frontline health workforce such as Lady Health Workers (LHWs). The National Urban Slum Profile indicates that lack of female health sector workforce participation is a “discouraging factor” for attendance of women and children at health facilities. The regular visits of LHWs in the slum communities are important because these health workers educate and promote healthy behaviour and provide basic curative healthcare services. The limited reach of Lady Health Workers (LHWs)is also confirmed by the findings from the coverage survey where in the sample of 14,491 mothers, it was found that just 44% (*n* = 6329) of the mothers reported knowing about the work of LHWs, and just 1% of mothers (*n* = 105) stated that the LHW provided information about vaccination. The primary function of the Lady Health Visitor (LHV) is to provide maternal and child health care. The survey of EPI facilities in eight cities found that 39% (*n* = 163) of EPI facilities do not have LHVs placed in them.

### Gender-friendly health services

The term “gender-friendly health services” is applied in the profiles in the context of facility design, including waiting room arrangements and water and sanitation infrastructure, and the degree to which these categories of infrastructure are gender-disaggregated.

Overall, 58% (*n* = 238) out of the 422 facilities surveyed in eight cities did not have gender-segregated waiting areas. Additionally, 43% (*n* = 180) of the 422 facilities did not have gender-segregated toilet areas for patients and staff, and 26% (*n* = 11) of facilities had no toilet facilities at all. Water supplies were available at only 65% (*n* = 276) of the 422 surveyed facilities. Gender-segregated waiting areas were available at 42% (*n* = 172) of the 422 surveyed facilities. Seven out of eight cities report inadequate seating capacity at clinics in or near slums and underserved areas. The National Urban Slum Profile indicated that the fact that 31% percent of the toilet facilities were gender mixed presented a “cultural barrier for females for easy use”, especially in cities such as Peshawar and Quetta where female caregivers may be considerably discouraged to get their children vaccinated due to the lack of such facilities [22]. Issues of privacy of examination rooms inside clinics were not examined in the profiles.

Availability of reproductive health services, female workforce participation and gender-friendly health infrastructure can also be classified as constituting “gender-friendly health services.” Presentation of findings will now extend beyond the health sector to include description and analysis of gender issues reflected in educational attainment, social connections and welfare service, as well as autonomy of decision making.

### Schools and health literacy

The urban slum profiles confirm that there are strong connections between a mother’s educational status and the coverage of vaccination in their children. These findings are attributable to several factors, including knowledge of mothers about the benefits of vaccination. In the national sample of 14,491 mothers of children aged 12-23 months, just 67% (*n* = 9659) could state that vaccination protects from diseases, with the remaining 33% (*n* = 4832)either not knowing or stating other reasons for vaccination being given.

A second reason is lack of access to school education. Overall, 27% of slums and underserved areas are without schools. Of the 1978 mothers in the national coverage survey who had children with zero doses of vaccines, 1505 (76%) had zero years of schooling, in contrast with the mothers of children of fully immunised children, of whom 47% (*n* = 3598) had zero years of schooling. The links between schooling and development is summed up in this extract from the qualitative discussions:

*“I still remember the day when I was married to him (my husband). I was 14 years old and in the 8th grade. I had ambitions of pursuing higher education and making something out of myself so that I could have a better life than my parents had and could choose a better life for my children-to-be. It seems it was not in the cards, after all.”(Resident Union Council 110, Punjab Province).*

There are two further criteria which can be applied relating to access to information about vaccination and modern health care. These include social connections and autonomy of decision making, both of which will be taken up in the following sections.

### Social connections

The Urban Slum profiles report widely on the availability of community-based and civil organisations in communities in eight of the ten cities, as well as on the number of formal and informal groups available in the slums and underserved communities, as outlined in Table 2.

**Table 2:** Prevalence of Community Organisations and Social Welfare Schemes in Slums and Underserved (U/S) Areas (*n* = 3114)

Types of Groups and Schemes in 3114 Urban Slum or Underserved Areas in Eight Cities		No.	% Availability in Slums & U/S Areas
Informal Groups	Masjid/Church, Zakat, School or Health Committees, Unregistered Community-Based Organisations, Jirga/Punchaiyat	1082	35%
Civil Society Organisation	Education, Health, Loans, Water, Human Rights	72	2.3%
Social Welfare Scheme	Loan and Stipend Schemes, Social Benefit Cards, Vocational Skill program.	1512	49%

The information demonstrates extremely low levels of formation of community level organisation and social connections with regards to health and education. These observations, coupled with the fact that only 6% of women surveyed work outside the home, suggest that lack of knowledge and demand and supply of services is not being compensated for through community organisation and social networks. It reinforces earlier findings of limited knowledge of households of the benefits of vaccination. It also raises questions about access of women to sufficient channels of communication enabling them to make autonomous and informed decisions about use of modern health and education services.

### Autonomy of decision making

**Fig. 3:**
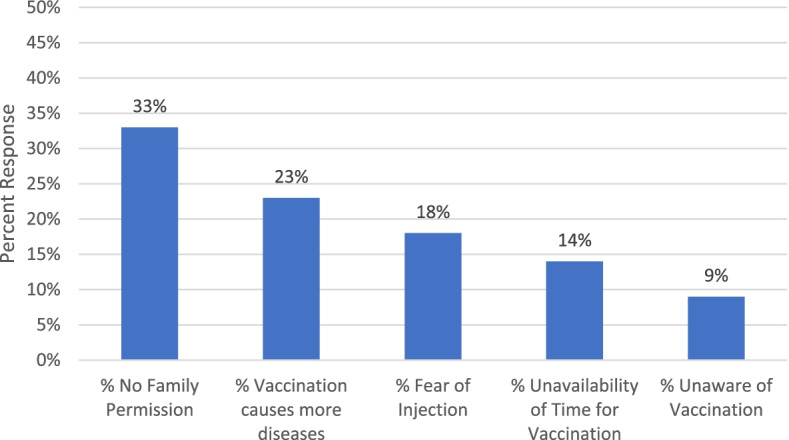
Mothers Reasons for Zero Dose of Children (*n* = 1985)

The immunization coverage survey in slums and underserved areas found that, of 14,441 mothers, 1985 of their children (14%) did not receive any vaccine. As illustrated in in Fig. 3, the most common response for why children did not receive vaccines was that family permission was not given (33%, *n* = 647)., with particularly high response rates for non-permission in the slums of Karachi (48%), and Quetta and Peshawar (both 43%). As Fig. 3 illustrates, there are also other reasons that include ‘fear of side effects’, ‘no time for vaccination’ and unaware of vaccination timings etc.

This lack of autonomy over decision making and limited social connections and availability of schools, coupled with low availability of a female health workforce participation, all interact to create a powerful social and institutional web of influence excluding women from informed decision making about health care. The following extracts from the Karachi urban slum profile highlight the challenges and complex dynamics of the urban poor women in seeking out health care:

*“It is very difficult to find time to go out of home. My husband takes care of responsibilities outside home…my husband does not allow me to travel alone and he does not have time available to take kids for vaccination by himself as it results in forgoing a day’s wage.*” *(Mother, Karachi City).*

*‘Bareera aged 12 months and 2 days, lives in a small, dilapidated slum …Residing here for the past 07 years, their house is composed of 02 rooms in which a total of 11 people live. ………. Aged 24, Bareera’s mother is illiterate and has not ever received any formal education. She is completely unaware of childhood vaccination and its significance. She says: “Bareera has not received any vaccination due to her grandfather’s disapproval. He becomes infuriated by such a proposition and is highly sceptical of the presence of men in vaccination centers….” (Mother, Slum area of Karachi) ’.*

From a perspective of inequities, autonomy of decision making not only relates to the issue of equal access of girls and women to vaccination services. The fact that a significant minority of boys are still not fully vaccinated demonstrates that a mother’s lack of autonomy in decision making affects access of both boys and girls to primary health care services. A plausible explanation for a significant proportions of boys not being fully vaccinated is related to the fear of side effects as illustrated in Fig. 3.

### Inequities in health and social outcomes

**Fig. 4:**
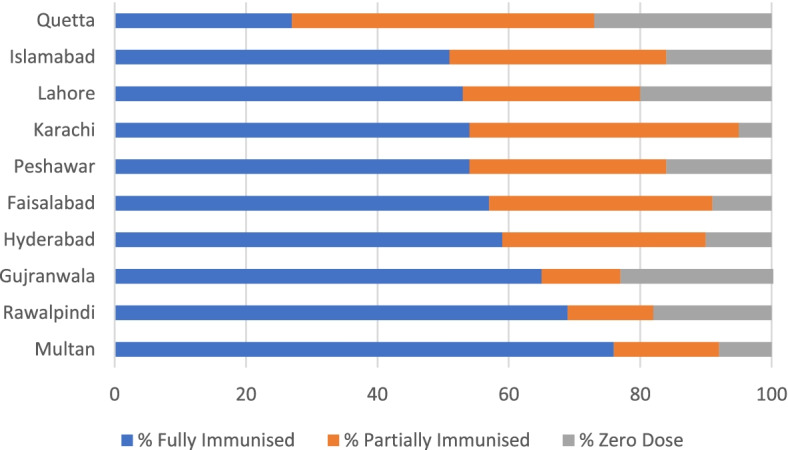
Percentage of fully Immunised, Partially Immunised and Zero Dose Children National Coverage Survey in Urban Poor Areas of Pakistan (14,531 Children aged 12-23 months)

Given the reported low availability of a female health workforce, health facilities and reproductive health services in the slums as reported in sections 4.1-4.3 of this paper, it would be reasonable to conclude that reproductive health and child mortality outcomes are likely to be comparable to PDHS findings in 2018. Although there are narrow gender gaps between fully immunised boys (54%) and fully immunised girls (53%) in the immunization coverage survey in slums and underserved areas, from a gender and public health impact perspective, the critical intervention for consideration is the role of women as primary health care givers supporting improved access of both girls and boys to immunization and other health services. Vaccination coverage rates are significantly lower in urban poor settings, with the Pakistan Demographic and Health Survey (PDHS) in 2018 demonstrating a coverage rate of 66% for fully immunised child rate compared to 54% (fully immunised child) in the urban health coverage survey [34]. Results from the immunisation coverage survey in 10 cities show that rates of fully immunised children (as verified by recall and card retention) range from 27% in Quetta to 76% in Multan Fig. 4.

## Discussion

### The Intersection of Social, Gender and Health Inequities

Equity remains central to Global Immunization Agenda 2030 and evidence shows that most of the zero dose children are found in communities where gender disparities are highly prevalent. Policy reviews [13], previous studies [11]and commentary [17] in Pakistan demonstrate that more research is needed to understand the complex interactions between various social forces that contribute to gender inequities in health care access and its eventual outcomes. As the findings from the urban slum profiles have demonstrated, complexity is illustrated in the multiple determinants of such inequities, that have included availability of health services, access to schooling, levels of health literacy, workforce participation and social connections, and autonomy of female decision making, all against a backdrop of socio-economic deprivation and the guiding narratives of gender norms.

These gender norms are characterised by inequitable power relations that disadvantage women in such areas as access to schooling, availability of reproductive health services and autonomy in decision making. Lack of social connections means that there are limited opportunities for gaining access to the required health information to make informed decisions, as well as limiting the development of the capabilities and social organisation to question gender based social disadvantage. Institutional policies and regulations that limit placements of schools, health facilities, social welfare support and a female health workforce in slums and underserved areas reinforces social disadvantage that is already being socialised in the urban poor communities.

Constraints on female workforce participation not only limits social connections and household incomes but have adverse effects on socio-economic development more generally. A clear linear relationship has been established in many countries between increased rates of female workforce participation and increased Gross Domestic Product (GDP) [35, 36], suggesting that there are significant opportunities in Pakistan to accelerate socio economic development through reducing gender inequities.

The findings of the Urban Slum Profiles are consistent with other findings on female workforce participation, which illustrate that, despite some growth in recent decades, female labour force participation in Pakistan is well below that of other countries with similar national incomes. An analysis of province-wide female workforce participation to population ratios in 2017-2018 found such rates were low in Balochistan (6.7%) and Khyber Pakhtunkhwa (9.3%) provinces, compared to rates in Sindh of 9.3% and in Punjab Province of 22.7% [37]. Low rates of female workforce participation are attributable in part to the persistence of gender norms, which identify males of the households as the major income earners and decision makers, resulting in the confining of women at home, or restricting female workforce participation to certain socially acceptable labour force roles. The persistence of these gender norms reduces workforce participation, which in turn reduces productivity and constrains household incomes [38].

Current cultural standpoints, social conditions, and institutional and policy settings means that current gender norms are being both socialised and institutionalised [26]. This demonstrates the ‘intersectionality’ of gender related health inequities, whereby it is the interaction between the broader social determinants of health and gender norms which are locking families into multi-generational cycles of health and social disadvantage [39].

### Health and social policy and planning implications

Given this level of intersectionality when considering gender and health, and in the light of the findings in this paper of overlapping and interacting gender and social disadvantage, what health policy and planning initiatives can be feasibly applied to negotiate a pathway through this complex maze of gender, health system and social determinants of health?

The Urban Slum profiles provide several recommendations in this regard, especially in relation to investment in a community-based female health workforce strategy. Although women constitute 56% of front-line workers in Pakistan [40], the main issue in the slums is the lack of absolute numbers of female health providers with capabilities to provide the full range of PHC services. Expanding the network of Lady Health Workers (LHWs) and Lady Health Visitors (LHVs) based in PHC facilities will bring services and health knowledge closer to communities in a gender-friendly manner, through expanded access to health knowledge, health outreach services and a female health workforce placed in EPI or renamed PHC facilities that are in or near the slums. This will require reforms to institutional policies, since, under current health administrative policies and arrangements, EPI facilities are mainly staffed by “vaccinators” [22] and are hence restricted in their capability to provide reproductive health care services.

Another potential point of entry into the complex web of the social and gender determinants of health in the slums is investment in civil society and community-based organisations, and informal groups, to provide viable and reliable communications to mothers, fathers and community leaders on the benefits of vaccination and reproductive health care services. This may also require reforms to institutional arrangements and policies of provincial and local governments to ensure that there is a favourable climate for building of such health partnerships and collaborations.

There are also longer-term institutional policies that will be required to support these directions which are clearly identified in the slum profiles, including development of urban health strategy and costed coverage plans, rebuilding of health infrastructure, improvements to housing and environmental conditions, advocacy for improved educational opportunities for girls and boys, and legal recognition of slums. Given the scale and longer-term nature of such developments, building of a female workforce and community-based social organisation to support health communications will in all probability be necessary conditions for ensuring there is adequate and capable social pressure to advocate for such longer-term social, institutional and policy change. Such reforms have the potential to be the precursor to a shifting of a “people’s narrative” [26] on gender norms, especially with regards to the role of women in health care and in employment outside the home more generally.

The idea of shifting of the of peoples’ and institutions’ narrative on social and gender norms demonstrates that, although feasible points of engagement are required to make earlier gains in reduction of gender related health inequities, in the longer-term an “ecological” strategic approach is required, that takes into account the multiple and overlapping determinants of health inequities that have been outlined in these urban studies [41]. An illustration of the importance of an ecological approach is demonstrated by the observation that the retention of women in the health workforce is influenced by the broader set of social and gender norms, with female health workers being required to “operate within the same gender systems that necessitate their appointment in the first place” [42]. It is for this very reason that that there needs to be a clear understanding of both social and health system dynamics to expand services for the most vulnerable or marginalised communities, which is consistent with the “whole of society approach” incorporating community engagement, multi-sector collaborations and public health as envisioned through global PHC frameworks [43]. The recommendations below highlight the importance of raising awareness of the role of social norms, household dynamics and health system readiness in together transforming the way we think about gender and health inequalities [9] Table 3.

**Table 3:** Selected Recommendations from a Global Review of Gender and Immunisation

• Assess context-specific household-decision making dynamics, and key influencers within communities to plan, implement and monitor immunization services for the most vulnerable • Leverage existing funding options to provide support for pro-gender demand & supply-side strategies, including strategies that support integration of immunisation with other reproductive and child health services • Explore Opportunities for gender transformative approaches aiming to achieve long-term change in the underlying social norms that drive gender inequality	

## Conclusion

The Urban Slum Profiles and immunization coverage survey in slums and underserved areas have found that the determinants of health and gender inequities are interacting and are having wide ranging impacts on PHC access for the urban poor. This being the case, the design of an initial short to medium term strategy will be critical to lay the ground for a longer-term ecological or PHC approach. The findings from these studies also provide an opportunity to develop a more gender and pro-equity focussed urban health strategy and costed action plans aligned with the national and provincial health plans, through the expansion of a female health workforce, in combination with institutional policies to support improved service availability and community organisation for PHC. Given the fact that over 13 million people are living in these urban poor areas in the 10 cities, the mainstreaming of gender into urban health strategy and operations has the potential to empower women towards more of a gender equal society, as well as improve access to essential health and social services. In doing so, such mainstreaming of gender policy demonstrates the potential to make a substantial contribution to the overall economic and social development of Pakistan.

## Data Availability

Refer to references [26], and [27].

## Footnotes

1 The 10 cities included Karachi and Hyderabad in Sindh Province, the five main cities of Punjab Province (Faisalabad, Gujranwala, Multan, Lahore, Rawalpindi), Quetta City in Balochistan Province, Islamabad Capital Territory and Peshawar in Khyber Pakhtunkhwa province.

2 The Union Council is the 5th and lowest level of Local Government in urban areas of Pakistan.
